# Information Theory in Formation Control: An Error Analysis to Multi-Robot Formation

**DOI:** 10.3390/e20080618

**Published:** 2018-08-20

**Authors:** Shuo Wan, Jiaxun Lu, Pingyi Fan, Khaled B. Letaief

**Affiliations:** 1 Tsinghua National Laboratory for Information Science and Technology (TNList), Department of Electronic Engineering, Tsinghua University, Beijing 100084, China; 2 Department of Electronic Engineering, Hong Kong University of Science and Technology, Hong Kong, China

**Keywords:** formation error, Bayes risk, mutual information, lower bound

## Abstract

Multi-robot formation control makes prerequisites for a team of robots to execute complex tasks cooperatively, which has been widely applied in both civilian and military scenarios. However, the limited precision of sensors and controllers may inevitably cause position errors in the finally achieved formation, which will affect the tasks undertaken. In this paper, the formation error is analyzed from the viewpoint of information theory. The desired position and the actually achieved position are viewed as two random variables. By calculating the mutual information between them, a lower bound of the formation error is derived. The results provide insights for the estimation of possible formation errors in the multi-robot system, which can assist designers to choose sensors and controllers with proper precision.

## 1. Introduction

In recent years, robots working in teams have been replacing complex single robots in both civilian and military applications. Such multi-robot systems can bring extraordinary benefits. When some robots do not work well, the problem may be fixed by simply replacing them with new ones. In this way, complex tasks are decomposed into multiple subtasks with relatively low complexity [1]. Therefore, the tasks can be accomplished with a lower cost. In military scenarios, autonomous robots are designed to cooperatively accomplish surveillance tasks [2] or spy on targets in adversarial regions. In civilian fields, automatic driving techniques may be helpful in intelligent transportation systems.

Formation control is a crucial technique for multi-robot systems. When executing a designated task, robots typically coordinate by maintaining sequential formations. For instance, when robots are required to move a large object, it is necessary for them to work in a certain formation [3]. In these situations, errors in the achieved formations may affect the tasks undertaken, which should be constrained in a reasonable range. Therefore, it is essential to estimate the formation error in system design. On the other hand, information theory has gained great success in estimation, which is a suitable choice to solve this problem.

There has been much work on multi-robot formation control. According to [4], existing results can mainly be categorized into position-, displacement- and distance-based control.

In position-based control, the absolute positions are measured by sensors with respect to a global coordinate system. According to the measurements, robots adjust their own positions to stand in a formation. To enhance the system robustness, interactions among robots were introduced to the controller. Furthermore, the strategy of feedback coordination was proposed in [5] to improve the stability.

In displacement-based control, robots sense neighboring companions and estimate their relative positions with respect to the global coordinate system. In this way, robots need to find their own orientation with respect to the desired formation. The global coordinate system itself and the absolute positions in it do not need to be explicitly known. Regarding this type of controller, the single-integrator modeled scenarios [6,7], the double-integrator modeled scenarios, the general linear agent scenarios [8] and the nonholonomic agent scenarios [9] were discussed recently in existing works.

In distance-based control, robots adjust distances between them to achieve the desired formation. In this process, each robot needs to sense neighboring companions and measure its relative positions with respect to its own coordinate system. The orientations of these local coordinate systems do not have to be unified. The distance-based control includes directed and undirected control. For undirected control, where the robot interaction graph is undirected, rigidity [10] was proposed as a requirement for the interaction graph. For directed control with directed interaction graphs, the notion of persistence was introduced to characterize the interaction graph [11]. Based on the basic feasibility requirements, problems of stabilization [12] and n-dimensional control [13] were further investigated.

The attitude control of robots is a supplement to position control in multi-robot systems. In [14], a general framework was presented to analyze the attitude tracking control for a rigid body. Furthermore, the attitude control for spacecraft [15,16] and leader-follower cooperative attitude control [17] were also investigated.

However, formation error analysis is still an open problem in theory. In multi-robot systems, formation error has to be constrained so that the tasks undertaken are not affected. Therefore, in system design, the error needs to be estimated for the adjustment of the system parameters. In the communication field, information theory has gained great success in error control. In multi-robot systems, it is also an appropriate choice to solve the estimation problem. In this paper, the formation error is defined in the form of distance. The Bayes risk representing formation error is estimated by calculating the mutual information between the measured value and the desired value. The results are further developed, and a lower bound of formation error is derived.

In this paper, our main focus is on leader-follower control scenarios, where each robot has a reference and follows it until the robot arrives at its desired position. Note that the leader traditionally refers to a leading robot followed by all other ones. However, in this paper, each robot is allowed to have a different reference as its leader. If the reference is chosen as other robots, it is a displacement-based control. If the reference is a fixed place, it is regarded as a position-based control. Each robot measures its reference with sensors and reaches the desired position by controllers. In this process, the sensors and controllers both result in errors. By introducing the notions of information theory, we derive a lower bound of formation error with respect to the precision of sensors and controllers. This newly-developed theoretical result can assist the design of a multi-robot formation system according to the application requirements. Finally, simulations are carried out to check our theoretical results.

For simplicity, the contributions in this paper are summarized as follows: (1) setting up a model to describe the errors of sensors and controllers in formation control; (2) applying information theory to estimate the formation error and deriving a lower bound with respect to system parameters; (3) testing the estimation results with a quasi-static control model in multi-robot systems.

The rest of the paper is organized as follows: In Section 2, the problem statement is provided. In Section 3, a problem model is set up. In Section 4, the issue is fit into a distributed estimation problem in information theory. The estimated error lower bound and its proof are provided in Section 5. Afterwards, the simulations are displayed to check the theoretical results in Section 6. Finally, the conclusion is given in Section 7.

## 2. Problem Statement

### 2.1. Basic Notations

Supposing there are *n* robots, each robot is denoted by a serial number i∈{1,2,…,n}. Their positions at time *t* are represented by $X\left(t\right)=\{x_{i}\left(t\right)|i=1,2,\ldots ,n\}$. Their initial positions $X\left(0\right)=\{x_{i}\left(0\right)|i=1,2,\ldots ,n\}$ are randomly distributed.

The formation is represented by $F=\left\{f_{1},f_{2},\ldots ,f_{n}\right\}$, where $f_{i}$ is the position of node *i* relative to the formation center. The origin of the coordinate system involving *F* is the formation center, which means $\sum_{i=1}^{n}f_{i}=0$.

In the multi-robot system, each robot is arranged according to a node in the formation. The arrangement is denoted by $D=\{d_{1},d_{2},\ldots ,d_{n}\}$, where $d_{i}\in \left\{1,2,\ldots ,n\right\}$ and $d_{i}=j$ means that robot *i* is designated to stand in position $f_{j}$. The optimal choice of *D* was investigated in [18,19].

According to arrangement *D*, each robot has a desired target denoted as $DS=\left\{ds_{1},ds_{2},\ldots ,ds_{n}\right\}$. The true targets of robots typically deviate from those in DS for errors from sensors. They are represented by $\hat{DS}=\left\{{\hat{ds}}_{1},{\hat{ds}}_{2},\ldots ,{\hat{ds}}_{n}\right\}$. The position error of robot *i* is Er(i). The overall formation error is denoted as Er.

### 2.2. Problem Definition

**Figure 1:**
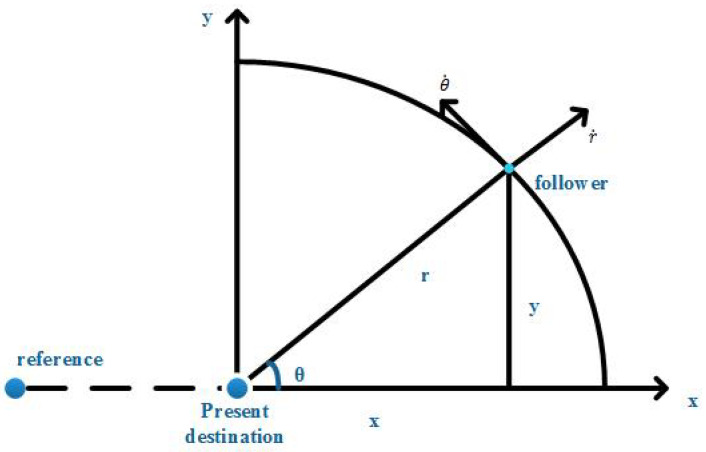
Leader follower control mode with a reference for each robot.

Considering *n* robots with random initial position X(0), they are supposed to achieve a formation *F*. A quasi-static model is taken, where the time is discretized. In each time slot, robots get the destination DS according to *D*, *F* and the positions of other robots. $\hat{DS}$ is the measurement of DS. With $\hat{DS}$, robots move towards their targets by controllers. After a certain amount of steps in such a way, the desired formation can finally be approached within a reasonable error range. The leader-follower mode is shown in Figure 1.

In the process, the measurement of DS may have errors brought by sensors. The controller may also bring errors to the final position. We mainly consider the combined error resulting from the elements mentioned above. Let Er(i) denote the position error of robot *i*. The formation error at time slot *t* is defined by:

(1) $$\mathrm{Er}\left(i\right)=\|x_{i}\left(t\right)-ds_{i}{\|}_{2}$$

From Er(i), the overall formation error is defined by (2).

(2) $$\mathrm{Er}=\frac{1}{n}\sum_{i=1}^{n}\mathrm{Er}\left(i\right)$$

From (2), it is obvious that the formation error is E[Er(i)]. That is to say, the overall formation error is the expectation of the position error on a single robot if every single robot plays the same role in the multi-robot formation system. Therefore, the objective of the problem is to estimate the error expectation E[Er(i)] for an arbitrary *i* with respect to the measuring error and controller precision.

## 3. Problem Model

The estimation of E[Er(i)] can be accomplished by information theory. In this section, the position is viewed as a random variable and modeled by a probability distribution. The factors bringing errors to positions are modeled mathematically. The model makes prerequisites for formation error analysis by the notions of information theory.

### 3.1. Dimensionality Reduction

The distance error of the position is the main concern, which is actually a one-dimensional variable. Then, for the analysis of two-dimensional formations, the dimensionality reduction is used by polar coordinates so that the distance model can be followed without lose of generality.

**Figure 2:**
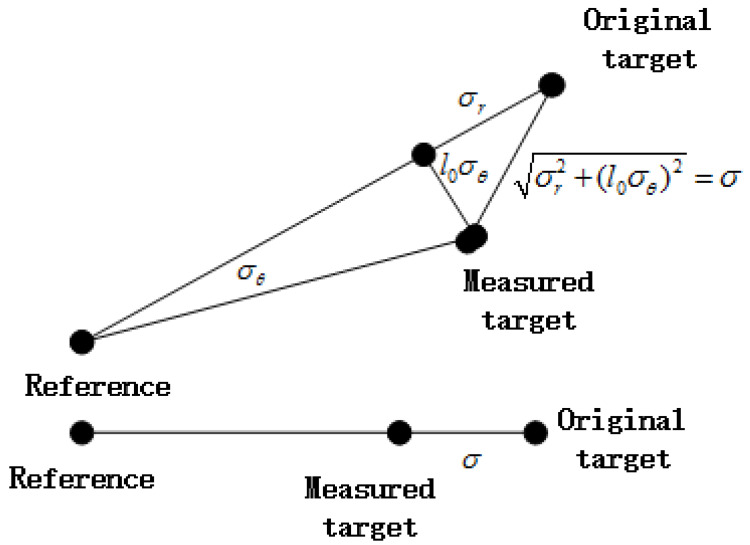
The dimensionality reduction performed on a two-dimensional formation.

As shown in Figure 2, in the polar coordinate system, the measured target has an angular deviation and a radius deviation. As the angular deviation is typically small, $\sigma_{\theta }$ is used to approximate sin($\sigma_{\theta }$). The radius is assumed to be within $\left[0,l_{0}\right]$, where $l_{0}$ is related to the formation size. By conservative estimation, the radius is considered to be $l_{0}$. The radius deviation is supposed to be $\sigma_{r}$. Then, as shown in Figure 2, the variance of the distance between DS and $\hat{DS}$ is $\sqrt{\sigma_{r}^{2}+{\left(l_{0}\sigma_{\theta }\right)}^{2}}$. Their corresponding measuring variance in polar coordinates is σ by using the project operation.

### 3.2. The Probability Distribution of Position

Supposing random variable *W* is the desired value of the distance relative to the reference, its corresponding probability distribution is $P_{W}$. To calculate the final mutual information, $P_{W}$ must first be modeled. In this system, supposing $W\in [0,l_{0}]$, an intuitive way is to consider $P_{W}$ as a uniform distribution. Note that it is more reasonable to consider the point uniformly distributed in an area of a disk. In this distance-based model, robot *i* is supposed to have an equal possibility to be located at each point in the disk with radius $l_{0}$. Therefore, the probability distribution is:

(3) $$P\left(W<r\right)=\frac{S_{r}}{S_{l_{0}}}=\frac{\pi r^{2}}{\pi l_{0}^{2}}=\frac{r^{2}}{l_{0}^{2}}\;\;\;\left(r<=l_{0}\right)$$

where P(W<r) represents the cumulative distribution function of $P_{W}$. $S_{r}$ and $S_{l_{0}}$ respectively represent the acreage of the disk with radius *r* and $l_{0}$.

The probability density function is given by:

(4) $$p_{W}\left(r\right)=\frac{dP(W<r)}{dr}=\frac{2r}{l_{0}^{2}}\;\;\;\left(r<=l_{0}\right)$$

### 3.3. Model of Sensor Error

As mentioned above, each robot needs to sense its reference and find the desired relative position as its target. The commonly-used sensors include monocular cameras, lasers or GPS. The distance between the measured target and the reference is the measured value of *W*, which is denoted by *X*. *W* and *X* are projected on a one-dimensional line. $P_{X|W}$ represents the distribution of measurement *X* conditioned on *W*. Typically in information theory, errors of the sensors are modeled by a conditional distribution. Here, we assume it to be a Gaussian distribution.

(5) $$P_{X|W=w}=\frac{1}{\sqrt{2\pi \sigma^{2}}}\times e^{\frac{{\left(X-w\right)}^{2}}{-2\sigma^{2}}}$$

where *w* is the value of *W* representing the mean value of the Gaussian distribution. σ is the measuring variance. Note that the target and the desired position may not be on the same line as the reference. Then, σ represents the combined error of the distance and angle, so that |X−W| is the distance between the target and desired position.

In the considered system, it is assumed that each robot measures the distance *n* times independently, following the same distribution. $X^{n}$ represents the results of all measurements. In this case, their joint distribution is $P_{X^{n}|W}$.

(6) $$P_{X^{n}|W=w}=\frac{1}{{\left(\sqrt{2\pi \sigma^{2}}\right)}^{n}}\times e^{\frac{\sum_{1}^{n}{\left(x_{i}-w\right)}^{2}}{-2\sigma^{2}}}$$

### 3.4. Model of Controller Error

**Figure 3:**
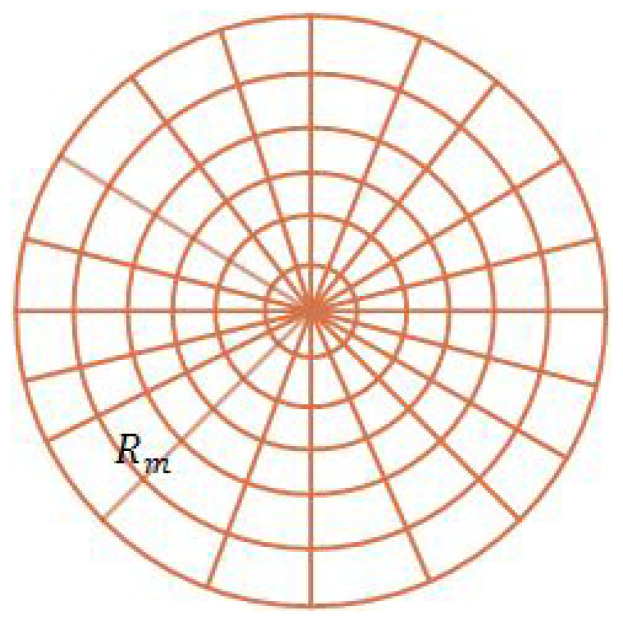
A partitioned disk with the reference as its center. The controller ensures precision in the discrete grids.

Apart from measuring errors, the operation of controllers may also result in errors. Given a certain precision of the controller, the deviation from the target is typically within a constant range. Therefore, each robot is supposed to be controlled to reach a near region around its designated position. That is to say, the controller only ensures precision in a discrete grid around the target. The leader-follower control model is based on a polar coordinate system. Then, as shown in Figure 3, it is assumed that the discrete grids are formed by equally dividing the angle and radius of a disk region. The radius of the disk is a constant set to be $R_{m}$, where $R_{m}\geq l_{0}$. Corresponding to the model, the measured value *X* is quantized to derive the final achieved value. Therefore, the operation precision of the controller is modeled by the quantization rate denoted by *b*. In quantization, the range $\left[0,R_{m}\right]$ is divided into $n_{r}$ regions, where $n_{r}$ satisfies:

(7) $$n_{r}=2^{b}$$

The distance error resulting from the operation of the controller is within the size $\frac{R_{m}}{2^{b}}$.

## 4. Lower Bound Estimation

### 4.1. Notions of Information Theory

A model of decentralized estimation in information theory is considered to analyze the formation error. In this model, the estimator has no direct access to the parameter of interest. However, it receives samples from a local sensor observing the parameter. After the samples are obtained, they are quantized and sent for analysis. Finally, an estimation of the parameter is obtained.

The quality of estimation is evaluated by distortion, which is defined by a function representing the deviation between two values. Bayes risk represents the minimum possible distortion derived from the best estimator. In [20], it gave lower bounds of the Bayes risk for the estimation problem.

**Figure 4:**
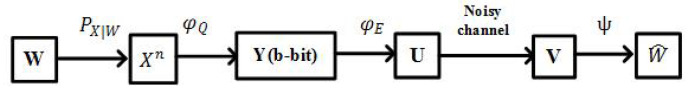
The model of decentralized estimation in information theory.

Figure 4 shows a decentralized estimation system. *W* is the parameter of interest, which is measured by local sensors. Let *W* be a random variable, the prior distribution is $P_{W}$. Given W=w, the sensor gets sample *X* generated from *W* by distribution $P_{X|W=w}$. Typically, the sensor gets *n* samples generated by distribution $P_{X^{n}|W=w}$. The samples are denoted as $X^{n}=\left\{X_{1},X_{2},\ldots ,X_{n}\right\}$. The samples $X^{n}$ are quantized by function $\varphi_{Q}$ into a *b*-bit message.

(8) $$Y=\varphi_{Q}\left(X^{n}\right)$$

*Y* is transformed into a codeword by $\varphi_{E}$.

(9) $$U=\varphi_{E}\left(Y\right)$$

*U* is transmitted through a noisy channel and received as *V*. Finally, the estimator calculates:

(10) $$\hat{W}=\psi \left(V\right)$$

as the estimation of *W*.

In multi-robot formation systems, the finally achieved position is actually an estimation of the exactly desired position. Therefore, the distributed estimation model in information theory is applied to analyze formation error. Before the estimation, the problem is fit into this model as follows:

1. *W* discussed above is the parameter of interest. It is the desired value of the distance.
2. $X^{n}$ are *n* independently drawn samples of *W*. The sensor on each robot measures the position *n* times. The *n* independent measurements are denoted as $\left\{x_{1},x_{2},\ldots ,x_{n}\right\}$.
3. The error resulting from the operation of the controller is modeled by the quantization of measurement $X^{n}$. The quantization rate is denoted as *b*.
4. The position achieved by control operation is the final result of multi-robot formation. Therefore, V=U=Y.

In this way, the distributed estimation model of information theory fits the estimation of formation error. Theories of information theory are applied to derive a lower bound on the Bayes risk of estimation. The results will guide the design of system parameters.

### 4.2. Lower Bound Estimation

As shown in Figure 4, the parameter of interest is *W*, which is derived from a prior distribution $P_{W}$. After sampling and quantization, the parameter is estimated as $\hat{W}=\psi \left(V\right)$. By using a non-negative distortion function $l:W\times W\to R^{+}$, the Bayes risk of estimation $\hat{W}$ is:

(11) $$R_{B}=\underset{\psi }{\inf}\;\;E\left(l\left(W,\psi \left(V\right)\right)\right)$$

where the distortion function *l* is of the form $l\left(w,\hat{w}\right)={\left\|w-\hat{w}\right\|}^{q}$. q≥1 and ‖.‖ is a norm. They can be specified according to the specific problem. In this paper, *W* represents the desired distance and $\hat{W}$ is the distance achieved by the operation of the controller. As mentioned above, the measured distance is projected on the same line with *W*, which makes it a one-dimensional problem. Therefore, function *l* is defined on $R^{1}$ with q=1, and $R_{B}$ represents the average error. In [20], it provided lower bounds on the Bayes risk. Based on the above model, an estimated lower bound of $R_{B}$ is obtained. This is also a lower bound of the final formation error.

**Theorem** **1.** *In multi-robot formation system, each robot measures its target n times. The measuring variance is σ. The error of the control operation represented by the quantization rate is b. If the desired distance satisfies $W\in [0,l_{0}]$, the lower bound of Bayes risk $R_{B}$ is:*

(12) $$R_{B}\geq \frac{l_{0}}{2e}\max\left\{2^{-(\log\frac{nl_{0}}{2\sigma }+\frac{1}{2}-\frac{1}{2}\log\left(2\pi e\right))},2^{-\left(1-e^{-\frac{nl_{0}^{2}}{2\sigma^{2}}}\right)b}\right\}$$

*where $l\left(w,\hat{w}\right)=\left|w-\hat{w}\right|$.*

**Remark** **1.** *$R_{B}$ represents the Bayes risk of the average position error of an arbitrary robot. The lower bound of $R_{B}$ is an estimated lower bound of E[Er(i)]. It is supposed that each robot plays the same role in the multi-robot formation system. Then, the expectation E[Er(i)] for an arbitrary i is the expectation on the overall formation error Er. Therefore, (12) is a lower bound of E[Er].*

## 5. Proof of Lower Bound

In this section, a proof of the lower bound in Theorem 1 is provided. In [20], the authors proposed theorems to estimate the lower bound of Bayes risk by information theory. The theorems are introduced as lemmas to analyze the formation error.

**Lemma** **1** ([20] (Theorem 3))**.** *For any arbitrary norm ‖·‖ and any q≥1, the parameter of interest $W\in R^{d}$ and W is distributed in [0,1] on each dimension. X are samples of parameter W. The Bayes risk on estimation of W with respect to the distortion function $l\left(w,\hat{w}\right)={\left\|w-\hat{w}\right\|}^{r}$ satisfies:*

(13) $$R_{B}\geq \underset{P_{U|W,X}}{\sup}\;\frac{d}{qe}{\left(V_{d}\Gamma \left(1+\frac{d}{q}\right)\right)}^{-\frac{q}{d}}\;2^{-\left(I(W;X|U)-h(W|U)\right)\frac{q}{d}}$$

*where $V_{d}$ is the volume of the unit ball in ($R^{d}$, ‖.‖) and Γ(.) is the gamma function. When a real-valued W is estimated with respect to $l\left(w,\hat{w}\right)=\left\|w-\hat{w}\right\|$, (13) is simplified as:*

(14) $$R_{B}\geq \underset{P_{U|W,X}}{\sup}\;\frac{1}{2e}\;2^{-\left(I(W;X|U)-h(W|U)\right)}$$

*Considering the unconditional version, there is a simpler form:*

(15) $$R_{B}\geq \frac{1}{2e}\;2^{-I(W;X)}$$

In fact, (15) is an unconditional version of Lemma 1, which gives a lower bound of $R_{B}$. This is for the case W∈[0,1]. If the range of the distribution is $\left[0,l_{0}\right]$, (15) is supposed to be multiplied by $l_{0}$. Besides, *W* and *X* are unconditional on *U*. Therefore, the unconditional version is taken for estimation.

**Lemma** **2** ([20] (Theorem 4))**.** *In decentralized estimation with a single processor, for any choice of $\phi_{Q}$ and $\phi_{E}$,*

(16) $$\begin{array}{c} I\left(W,V\right)\leq \min\{I\left(W,X^{n}\right)\eta_{T},\eta \left(P_{X^{n}},P_{W|X^{n}}\right)\left(H\left(X^{n}\right)\wedge b\right)\eta_{T},\\ \eta (P_{X^{n}},P_{W|X^{n}})CT\} \end{array}$$

*where C is the Shannon capacity of the noisy channel $P_{V|U}$ and $\eta_{T}$ represents the properties of the channel.*

In this problem, there is no process across the noisy channel. Then:

(17) $$\eta_{T}=1$$

$\eta (P_{X^{n}},P_{W|X^{n}})$ is defined as follows. Given a channel with conditional distribution *K* whose input alphabet is *X* and output alphabet is *Y*, the reference input distribution on *X* is μ. D(.|.) is the K-L divergence. For a constant c∈[0,1) and any other input distribution ν on *X*, if D(νK‖μK)≤cD(ν‖μ), *K* satisfies strong data processing inequalities (SDPI) at μ.

The SDPI constant of *K* at input distribution μ is defined as:

(18) $$\eta \left(\mu ,K\right)=\underset{\nu :\nu \neq \mu }{\sup}\frac{D\left(\nu K\|\mu K\right)}{D\left(\nu \|\mu \right)}$$

The SDPI constant of *K* is defined as:

(19) $$\eta \left(K\right)=\underset{\mu }{\sup}\;\eta \left(\mu ,K\right)$$

With Lemma 2, an upper bound of I(W,V) is derived with respect to the system parameters. Lemma 1 gives a lower bound of Bayes risk with respect to I(W,V). Therefore, the combination of Lemmas 1 and 2 leads to a final estimation of the lower bound.

In this problem, the channel loss in information theory is not considered, for there is no process across the channel. That is, the channel capacity *C* is infinite. In (16), the upper bound of I(W,V) is the minimum value of three elements. Among them, $\eta (P_{X^{n}},P_{W|X^{n}})CT$ is related to *C*. Therefore, it is supposed to be ignored. Then, the upper bound (16) is simplified as:

(20) $$I\left(W,V\right)\leq \min\{I\left(W,X^{n}\right),\eta \left(P_{x^{n}},P_{W|X^{n}}\right)\left(H\left(X^{n}\right)\wedge b\right)\}$$

In formation error analysis, $X^{n}$ denotes *n* measurements of parameter *W*. Each of its element $X_{i}$ satisfies $X_{i}\in \left[0,l_{0}\right]$. $X_{i}$ is a continuous random variable. After the quantization with rate *b*, there will be loss of information. Therefore, $H(X^{n})$ is larger than *b*. That is,

(21) $$H(X^{n})\wedge b=b$$

Then, the upper bound (20) is further simplified as (22).

(22) $$I\left(W,V\right)\leq \min\{I\left(W,X^{n}\right),\eta \left(P_{X^{n}},P_{W|X^{n}}\right)b\}$$

Then, the following step is to estimate $I(W,X^{n})$ and $\eta (P_{x^{n}},P_{W|X^{n}})b$ separately.

Clarke [21] showed that:

(23) $$\begin{array}{c} I\left(W,X^{n}\right)=\frac{d}{2}\log\left(\frac{n}{2\pi e}\right)+h\left(W\right)+\frac{1}{2}E\left[\log\;\det\;J_{X|W}\left(W\right)\right]\\ +o\left(1\right) \end{array}$$

where h(W) represents the differential entropy of *W*. $J_{X|W}\left(W\right)$ is the Fisher information matrix.

From (4), the probability distribution density of *W* is $p_{W}\left(r\right)=\frac{2r}{l_{0}^{2}}$.

Then, the differential entropy of *W* is:

(24) $$\begin{array}{cc} h(W) & =E(-\log\left(p_{W}\left(r\right)\right))\\ & =\int_{0}^{l_{0}}-\frac{2r}{l_{0}^{2}}\;\log\left(\frac{2r}{l_{0}^{2}}\right)\;dr\\ & =\frac{1}{2}-\log\frac{2}{l_{0}} \end{array}$$

The Fisher information is:

(25) $$\det\;J_{X|W}\left(W\right)=-E\left[\frac{\partial^{2}}{\partial W^{2}}\;\log\left(P_{X|W}\right)\right]$$

From (6), P(X|W) is a joint Gaussian distribution of *n* independent samples. Then:

(26) $$\log\left(P_{X|W}\right)=-\frac{n}{2}\log\left(2\pi \sigma^{2}\right)-\frac{1}{2\sigma^{2}}\sum_{i=1}^{n}{\left(x_{i}-W\right)}^{2}$$

Substituting (26) into (25),

(27) $$\begin{array}{cc} \det\;J_{X|W}\left(W\right) & =-E[\frac{\partial^{2}}{\partial W^{2}}\left(-\frac{1}{2\sigma^{2}}\sum_{i=1}^{n}{\left(x_{i}-W\right)}^{2}\right)]\\ & =\frac{1}{2\sigma^{2}}E\left[\frac{\partial }{\partial W}\left(-2\sum_{i=1}^{n}\left(x_{i}-W\right)\right)\right]\\ & =\frac{1}{2\sigma^{2}}E\left[2n\right]\\ & =\frac{n}{\sigma^{2}} \end{array}$$

In this system, since $W\in R^{1}$, d=1. By (23) and (24), the estimation of $I(W,X^{n})$ is:

(28) $$\begin{array}{cc} I\left(W,X^{n}\right) & =\frac{1}{2}\log\left(\frac{n}{2\pi e}\right)+\frac{1}{2}-\log\left(\frac{2}{R}\right)+\frac{1}{2}\log\left(\frac{n}{\sigma^{2}}\right)+o\left(1\right)\\ & =\log\;\frac{nR}{2\sigma }+\frac{1}{2}-\log\;2\pi e+o\left(1\right) \end{array}$$

As for the estimation of $\eta (P_{x^{n}},P_{W|X^{n}})b$, the critical step is to estimate $\eta (P_{X^{n}},P_{W|X^{n}})$. Here, a lemma in [20] is introduced to achieve it.

**Lemma** **3** ([20] (Lemma 5))**.** *For a joint distribution $P_{W,X}$, suppose there is a constant α∈(0,1] such that the forward channel $P_{X|W}$ satisfies:*

(29) $$\frac{dP_{X|W=w}}{dP_{X|W=w^{{}^{\prime }}}}\left(x\right)\geq \alpha$$

*for all x∈X and $w,w^{\prime }\in W$.*

*Then, the SDPI constants of the forward channel $P_{X|W}$ and the backward channel $P_{W|X}$ satisfy:*

(30) $$\eta \left(P_{X|W}\right)\leq 1-\alpha$$

*and:*

(31) $$\eta \left(P_{W|X}\right)\leq 1-\alpha$$

The measurement of parameter *W* is $X^{n}$. Then, $\eta \left(P_{W|X}\right)$ is replaced with $\eta \left(P_{W|X^{n}}\right)$. From (19),

(32) $$\eta \left(P_{W|X^{n}}\right)\geq \eta \left(P_{X^{n}},P_{W|X^{n}}\right)$$

Then, together with (31),

(33) $$\begin{array}{cc} \eta (P_{X^{n}},P_{W|X^{n}}) & \leq \eta \left(P_{W|X^{n}}\right)\\ & \leq 1-\alpha \end{array}$$

Therefore, the critical step is to estimate α.

From (6), $P_{X|W=w}$ is a joint Gaussian distribution, with *w* as its mean. Then:

(34) $$\begin{array}{cc} \frac{dP_{X|W=w}}{dP_{X|W=w^{\prime }}}\left(x\right) & =\frac{\frac{1}{{\left(\sqrt{2\pi \sigma^{2}}\right)}^{n}}e^{\sum_{i=1}^{n}-\frac{{\left(x_{i}-w\right)}^{2}}{2\sigma^{2}}}}{\frac{1}{{\left(\sqrt{2\pi \sigma^{2}}\right)}^{n}}e^{\sum_{i=1}^{n}-\frac{{\left(x_{i}-w^{\prime }\right)}^{2}}{2\sigma^{2}}}}\\ & =e^{\sum_{i=1}^{n}\frac{{\left(x_{i}-w^{\prime }\right)}^{2}}{2\sigma^{2}}-\sum_{i=1}^{n}\frac{{\left(x_{i}-w\right)}^{2}}{2\sigma^{2}}}\\ & =e^{\frac{\sum_{i=1}^{n}\left(w-w^{\prime }\right)\left(2x_{i}-w-w^{\prime }\right)}{2\sigma^{2}}} \end{array}$$

From (29), α is the minimum value of (34). As each element in $X^{n}$ is independent of each other, the problem is equivalent to minimizing $\left(w-w^{\prime }\right)\left(2x_{i}-w-w^{\prime }\right)$. Let:

(35) $$h=\min\left\{\left(w-w^{\prime }\right)\left(2x-w-w^{\prime }\right)\right\}$$

where $x\in [0,l_{0}]$ and $w,w^{\prime }\in \left[0,l_{0}\right]$. (35) is transformed as:

(36) $$\left(w-w^{\prime }\right)\left(2x-w-w^{\prime }\right)=2\left(w-w^{\prime }\right)x-\left(w^{2}-w{}^{\prime }{}^{2}\right)$$

When $w>w^{\prime }$, (36) increases with *x*. Therefore, (36) is minimized at x=0. Then:

(37) $$h=\min\left\{-\left(w^{2}-w{}^{\prime }{}^{2}\right)\right\}\;\left(w>w^{\prime }\right)$$

As $w,w^{\prime }\in \left[0,l_{0}\right]$,

(38) $$h=-l_{0}^{2}\;\left(w=0,w^{\prime }=l_{0}\right)$$

Similarly, when $w<w^{\prime }$, (36) decreases as *x* increases. Therefore, (36) is minimized at $x=l_{0}$. Then:

(39) $$h=\min\{2\left(w-w^{\prime }\right)l_{0}-\left(w^{2}-w{}^{\prime }{}^{2}\right)\}$$

To derive the minimum point, (36) is transformed as follows:

(40) $$h=\min\{w{}^{\prime }{}^{2}-2w^{\prime }l_{0}-w^{2}+2wl_{0}\}$$

Equation (40) is a quadratic function of $w^{\prime }$ and $w<w^{\prime }$. Then, the minimum value is reached at $w^{\prime }=l_{0}$. That is:

(41) $$h=\min\left\{-l_{0}^{2}-w^{2}+2wl_{0}\right\}\;\left(w^{\prime }=l_{0}\right)$$

Equation (41) is a quadratic function of *w*. The minimum point of (41) is w=0. Therefore:

(42) $$h=-l_{0}^{2}\;\left(w=0\right)$$

Together with (38) and (42),

(43) $$\min\left\{\left(w-w^{\prime }\right)\left(2x-w-w^{\prime }\right)\right\}=-l_{0}^{2}$$

Then:

(44) $$\frac{\sum_{i=1}^{n}\left(w-w^{\prime }\right)\left(2x_{i}-w-w^{\prime }\right)}{2\sigma^{2}}\geq -\frac{nl_{0}^{2}}{2\sigma^{2}}$$

Together with (34),

(45) $$\frac{dP_{X|W=w}}{dP_{X|W=w^{\prime }}}\left(x\right)\geq e^{-\frac{nl_{0}^{2}}{2\sigma^{2}}}$$

That is (see (29)):

(46) $$\alpha =e^{-\frac{nl_{0}^{2}}{2\sigma^{2}}}$$

Finally, from Lemma 3 and (33),

(47) $$\eta \left(P_{X^{n}},P_{W|X^{n}}\right)\leq 1-e^{-\frac{nl_{0}^{2}}{2\sigma^{2}}}$$

With the estimated value of $I(W,X^{n})$ and $\eta (P_{x^{n}},P_{W|X^{n}})b$, the conclusion is:

(48) $$\begin{array}{c} I\left(W,V\right)\leq \min\left\{\log\frac{nl_{0}}{2\sigma }+\frac{1}{2}-\frac{1}{2}\log\left(2\pi e\right),\left(1-e^{-\frac{nl_{0}^{2}}{2\sigma^{2}}}\right)b\right\} \end{array}$$

In (15), I(W,X) is supposed to be replaced with I(W,V), for *W* is estimated by *V* here. The lower bound in (15) is multiplied by parameter $l_{0}$. According to the upper bound given in (48), the final lower bound of the Bayes risk is derived as:

(49) $$R_{B}\geq \frac{l_{0}}{2e}\max\left\{2^{-(\log\frac{nl_{0}}{2\sigma }+\frac{1}{2}-\frac{1}{2}\log\left(2\pi e\right))},2^{-\left(1-e^{-\frac{nl_{0}^{2}}{2\sigma^{2}}}\right)b}\right\}$$

Therefore, the proof of Theorem 1 is accomplished.

## 6. Simulations

To test the formation error analysis result, the leader-follower mode to achieve a formation is first realized in MATLAB.

In leader-follower mode, there are plenty of controllers with different operation precisions. The controllers may use operations with complex techniques to control the acceleration of movements. In fact, the moving speed can be viewed as a constant during a short time interval. Therefore, the single-integrator controller in (50) is applied to represent other controllers in a quasi-static model. Note that in this control model, each movement represents the average movements in a time slot. In this way, the controller is simplified, and the simulation concentrates on testing the formation error analysis with respect to system parameters.

(50) $$\dot{x_{i}}=u_{i}\;\;\;\left(u_{i}\leq u_{max}\right)$$

In (50), $u_{i}$ is the control input and $x_{i}$ is the position. $u_{i}$ is the differential derivative of $x_{i}$. $u_{max}$ is the maximum speed of robots. The direction of $u_{i}$ is not restricted. That is, robots are supposed to be omnidirectional. In simulations, the continuous control operation is replaced by the discrete control model. Time is discrete, and the refreshment of speed input is also discrete. In the quasi-static model, robots are supposed to update the speed instantaneously. In each time slot, each robot will move a grid towards its target. Then, after several time slots, each robot will reach its target grid, and the system will reach a stable state, which ends the whole process. The grids mentioned above are shown in Figure 3. The controllers are set to ensure precision in discrete grids. If the robot discovers that there are any other robot in its target grid or heading towards the grid, it will wait a time slot until the grid is clear to move in it. In this way, collisions are avoided in the process.

### 6.1. Formation Illustration

**Figure 5:**
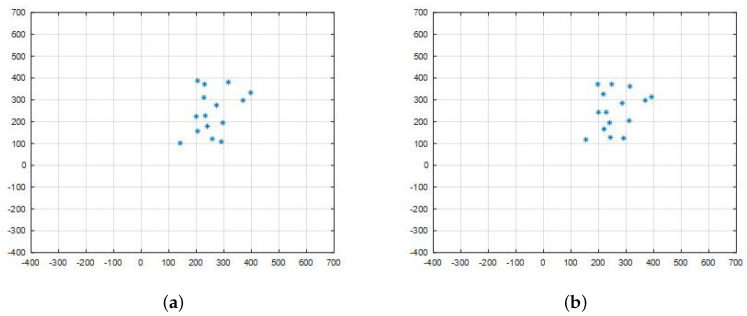

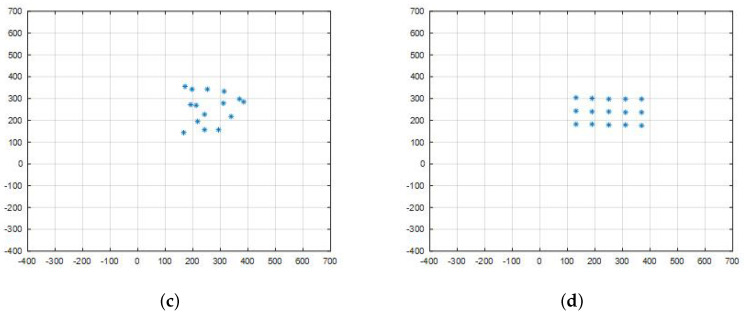
(**a**) shows the robots with randomly deployed initial positions. (**b**,**c**) show that the robots are moving in leader-follower mode. (**d**) shows that the robots reach a square formation.

In simulations, the working space is a square area with size 1100×1100. There are 15 robots, and the initial positions are generated randomly. The controlling range $R_{m}$ is set as 300, and the quantification rate is b=7. That is, the operation error of the controller is within $\frac{R_{m}}{2^{b}}$. The maximum speed $u_{max}$ is five, and the measuring variance is σ=1. As shown in Figure 5, robots in random initial positions are required to reach a square formation under the quasi-static control mode.

### 6.2. Formation Error Analysis

To investigate the offsets of the theoretical lower bound on formation error, we carry out simulations to check the errors of achieved formations with respect to the system parameters. The experimental data are fit with the least squares method. The calculated lower bound is also shown for comparison.

**Figure 6:**
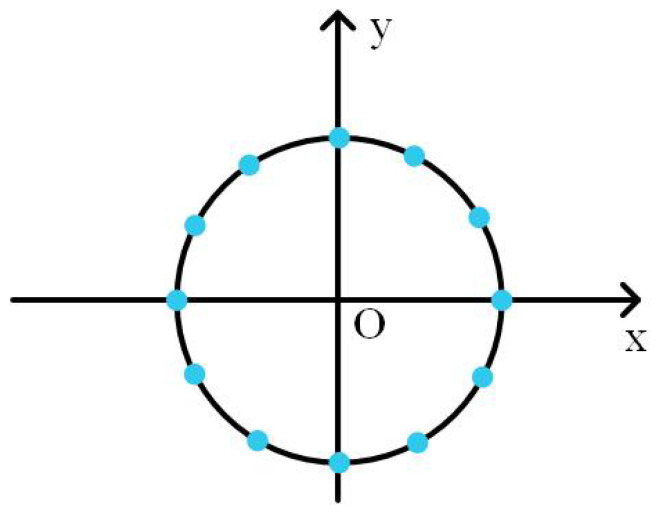
The circle formation in the simulation test. Each node represents a position.

In each simulation, the conditioning parameter is adjusted in a previously set range. For each parameter value, 20 experiments are carried out. Robots are in random initial positions. They achieve the circle formation shown in Figure 6. The formation error for each experiment is recorded.

#### 6.2.1. Measuring Times *n*

As discussed above, each robot measures the desired position *n* times to get its final target. There are 15 robots. The one-dimensional measuring variance σ is set to be two, and $\sigma_{\theta }=10^{-5}$. The controlling range $R_{m}$ is 200. The distance is partitioned into 200 sections within which the precision of control operation is ensured. Converting it into the quantization rate, it is b=7.64. The maximum speed $u_{max}$ is five. *n* ranges from 1–60. In the theoretical analysis, the distance empirical ranging threshold is $l_{0}=120$.

**Figure 7:**
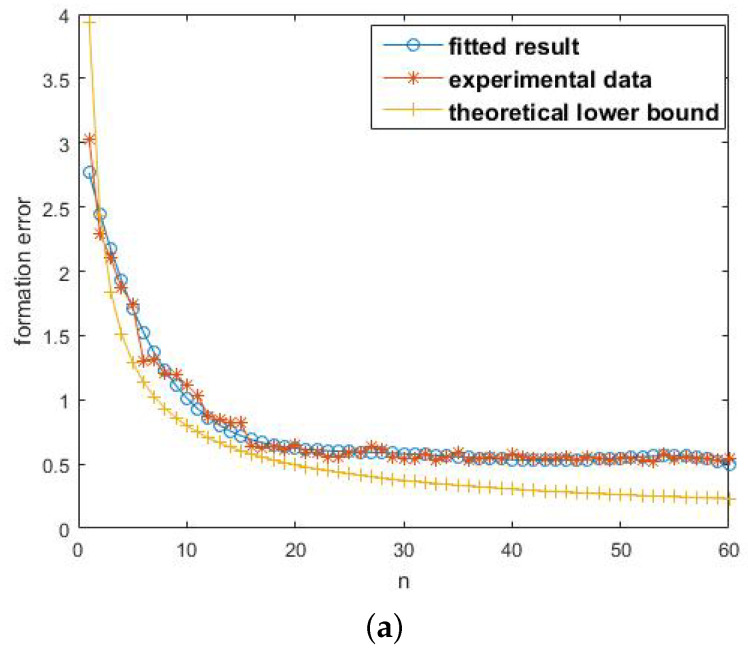

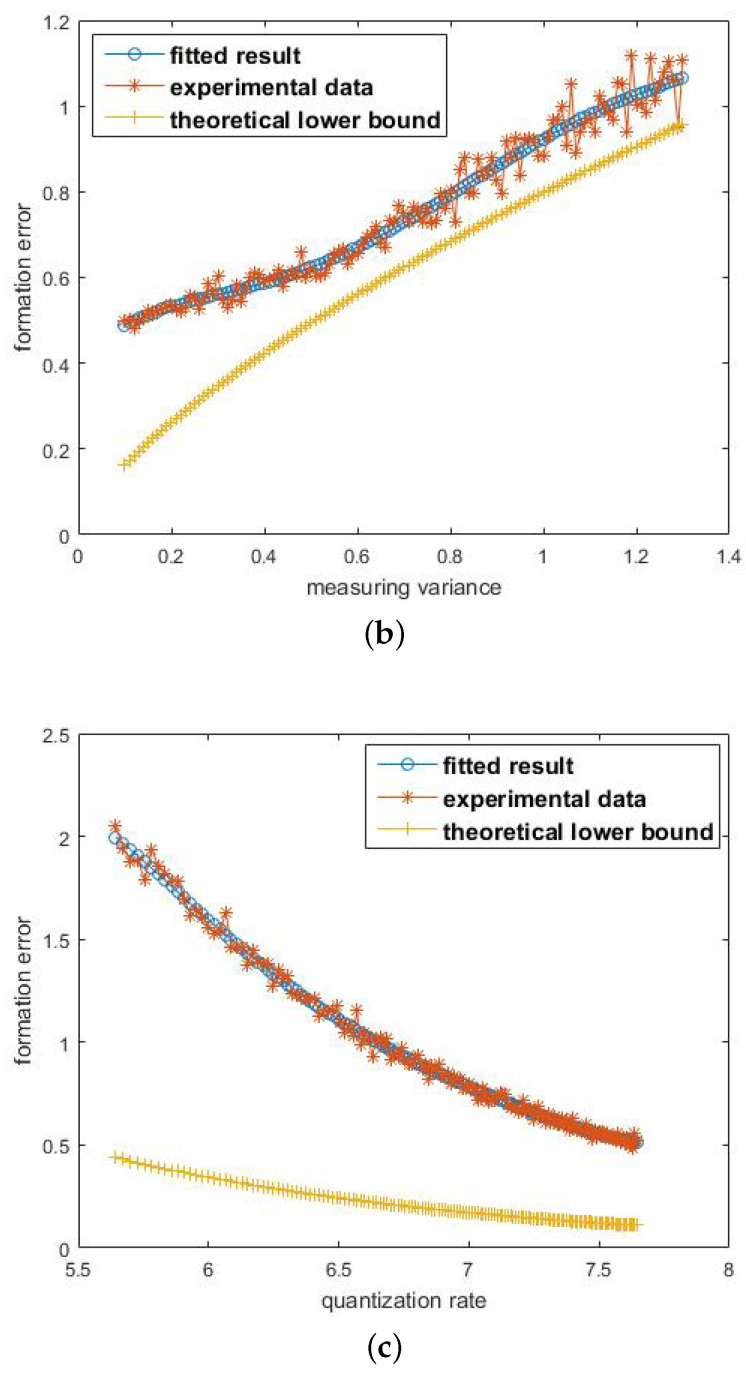
(**a**) shows the tendency of formation errors with respect to *n*. It depicts the experimental data, the fitted curve and the calculated lower bound. (**b**) shows the tendency of formation errors with respect to measuring variance σ. (**c**) shows the tendency of formation errors with respect to the quantization rate *b*.

Figure 7a shows the formation error with respect to *n*. All curves decrease as *n* increases. As a matter of common sense, when *n* is small, increasing *n* apparently reduces the formation error. When *n* is large enough, increasing *n* does not obtain obvious evolution. The tendency of curves is consistent with common sense. The curve marked by “*” represents the experimental data, and the curve marked by “o” is the fitting result with the least squares method. The curve marked by “+” is the calculated lower bound by Theorem 1. At the beginning, the theoretical result is a little larger than the experimental data. This is due to less statistics performed. Note that in the remaining majority part, the lower bound is below the experimental data, and the tendency is the same.

#### 6.2.2. Measuring Variance σ

There are 15 robots each of which measures the distance 10 times. The controlling range $R_{m}$ is 200. The distance is partitioned into 200 sections within which the precision of control operation is ensured. Converting it into the quantization rate, it is b=7.64. The maximum speed $u_{max}$ is five. The one-dimensional variance σ ranges from 0.1–1.3, and $\sigma_{\theta }=10^{-5}$. In the theoretical analysis, the distance empirical ranging threshold is $l_{0}=120$.

Figure 7b shows the tendency of formation error with respect to measuring variance σ. In general, all curves increase with σ. As a matter of common sense, when the measuring variance increases, the measuring error increases and therefore results in the increase of formation errors. The tendency of curves is consistent with the common intuition. The theoretical lower bound is below the experimental data, and their tendencies are the same.

#### 6.2.3. Quantification Rate *b*

There are 15 robots each of which measures the distance 10 times. The controlling range $R_{m}$ is 200, and the maximum speed $u_{max}$ is five. The one-dimensional measuring variance of each robot is σ=0.1 and $\sigma_{\theta }=10^{-5}$. The whole range is partitioned into $2^{b}$ grids within which the precision of control operation is ensured. The quantization rate *b* satisfies b∈[5.64,7.64]. In the theoretical analysis, the distance empirical ranging threshold is $l_{0}=120$.

Figure 7c shows the tendency of formation error with respect to the quantization rate *b*. All curves decrease as *b* increases. As a matter of common sense, when *b* increases, the operation precision of the controller increases, resulting in the decrease of formation errors. When *b* is large enough, the decreasing tendency slows down. The tendency of curves is consistent with the common intuition. The lower bound is below the experimental data. Though there is a gap between them, their tendencies are similar.

## 7. Conclusions

In this paper, the problem of formation error analysis was figured out with information theory. The formation error was defined by distance and the error analysis issue was transformed into an estimation problem. Then, by models in information theory, a lower bound was derived, which could assist in choosing proper sensors and controllers in system design. In the simulations, the formation error was recorded and compared with the estimated lower bound.
